# ADHD characteristics: I. Concurrent co-morbidity patterns in children & adolescents

**DOI:** 10.1186/1753-2000-2-15

**Published:** 2008-07-03

**Authors:** Josephine Elia, Paul Ambrosini, Wade Berrettini

**Affiliations:** 1 Department of Child and Adolescent Psychiatry, The Children's Hospital of Philadelphia, Philadelphia, PA, USA; 2 Department of Psychiatry, The University of Pennsylvania, Philadelphia, PA, USA; 3 Department of Psychiatry, Drexel University College of Medicine, Philadelphia, PA, USA

## Abstract

**Objective:**

342 Caucasian subjects with attention deficit/hyperactivity disorder (ADHD) were recruited from pediatric and behavioral health clinics for a genetic study. Concurrent comorbidity was assessed to characterize the clinical profile of this cohort.

**Methods:**

Subjects 6 to 18 years were diagnosed with the Schedule for Affective Disorders & Schizophrenia for School aged Children (K-SADS-P IVR).

**Results:**

The most prevalent diagnoses co-occurring with ADHD were Oppositional Defiant Disorder (ODD) (40.6%), Minor Depression/Dysthymia (MDDD) (21.6%), and Generalized Anxiety Disorder (GAD) (15.2%). In Inattentive ADHD (n = 106), 20.8% had MDDD, 20.8% ODD, and 18.6% GAD; in Hyperactive ADHD (n = 31) 41.9% had ODD, 22.2% GAD, and 19.4% MDDD. In Combined ADHD, (n = 203), 50.7% had ODD, 22.7% MDDD and 12.4% GAD. MDDD and GAD were equally prevalent in the ADHD subtypes but, ODD was significantly more common among Combined and Hyperactive ADHD compared to Inattentive ADHD. The data suggested a subsample of Irritable prepubertal children exhibiting a diagnostic triad of ODD, Combined ADHD, and MDDD may account for the over diagnosing of Bipolar Disorder.

**Conclusion:**

Almost 2/3^rd ^of ADHD children have impairing comorbid diagnoses; Hyperactive ADHD represents less than 10% of an ADHD sample; ODD is primarily associated with Hyperactive and Combined ADHD; and, MDDD may be a significant morbidity for ADHD youths from clinical samples.

## Background

The primary goal of this report is to characterize the concurrent comorbidy patterns identified in a clinical sample of ADHD subjects recruited for this genetic study. If more homogenous comorbid ADHD subtypes are identified, there is the potential for their validation through their genetic characteristics. In any assessment of psychopathology using standardized diagnostic interview schedules, comorbidity is the rule however it is infrequently considered in genetic studies. Concurrent comorbidity means the disorders are present at the same time of assessment as opposed to lifetime comorbidity which implies at any time over one's life. Comorbidity also can be classified into two broad patterns, homotypic or heterotypic [1]. These characteristics refer, respectively, to the continuity of a diagnostic pattern or the emergence of different diagnostic patterns over a lifetime. For ADHD these types of comorbidity could be represented by residual ADHD in adults or the emergence of an affective syndrome at some time in the course of ADHD. The relevance of these issues for the present study is that the focus of the genetic analysis of ADHD must consider the comorbid patterns of ADHD itself. It may be that the "true" syndrome is actually reflected by a specific comorbid pattern [2]. Only by more clearly defining the ADHD construct will there be any success in identifying true genetic/biological markers for this disorder [3].

Reports of comorbidity in ADHD include both community and clinic samples. These are not compatible patient pools because of the biased referral patterns and the increased rates of disorder in any clinical group. Furthermore, only with a general population sample can a true base rate of a disorder be calculated and comorbid excessive identified [2]. Reported comorbid rates in ADHD are substantial [4] yet prevalence figures frequently reflect epidemiological samples or life times rates within clinical groups. Other large clinical samples such as the MTA study [5] only included those with ADHD combined subtype ages 7 to 9.9. Nonetheless over 2/3^rd ^of the MTA sample had concurrent comorbid disorders identified with a parental DISC interview.

Angold et al's [1] extensive review of community studies of comorbidity quantify the excess rates of comorbid conditions in ADHD by calculating the odds ratio of the major co-occurring disorders. They noted that the odds ratios for ADHD-ODD/CD, ADHD-depression, and ADHD-anxiety were 10.7, 5.5, and 3.0 respectively, suggesting that comorbidity is not due to referral bias that might be expected in clinical samples. Mood disorders may be high in preadolescent ADHD regardless of referral source [6] and this may be a heterotypic comorbid pattern particularly in girls [7]. However, case ascertainment, definitions and assessment methodology, and comparison control groups chosen must be considered in order to understand the true significance of these findings.

Comorbidity rates are dependent on the assessment methodology. In assessing ADHD children Biederman's group [6,8] has relied on the K-SADS-E [9] that measures current and life time disorders. The DISC utilized in the MTA study [10] measures past 6 months for all disorders except for CD which is for 12 months. The DICA measures current and lifetime rates; however this distinction is not differentiated in published reports [11,12]. Various symptom rating scales such as the CBCL (rates symptoms now or within past 6 months) or the Devereux Scales of Mental Disorders (measures symptoms during past four weeks) [13] have also been utilized to evaluate ADHD comorbidity. Some clinical studies have used combinations of diagnostic interviews and rating scales [14,15]. Adult and follow-up studies of ADHD have followed a similar pattern using combinations of diagnostic and rating instruments which again assess current, lifetime, or time specific intervals. Introducing genetic data to validate comorbid ADHD subtypes therefore, must be clear about the qualitative aspect of the comorbid data set. The genetic associations thus may be on a heterotypic/homotypic or concurrent/lifetime pattern.

## Methods

### Participants

This report describes participants from an ongoing ADHD genetic study aiming to recruiting 500 parents/child triads with one or more ADHD probands. The sample currently consists of 342 children and adolescents from 302 families (33 families with 2 siblings and 6 families with 3 siblings for a total of 342 children). All subjects were North-American of European descent. Other groups were excluded because haplotype frequencies can vary substantially across major world populations [16] lowering power of the study to detect genetic association if multiple groups were included. This protocol was approved by the Institutional Review Boards of The Children's Hospital of Philadelphia and the University of Pennsylvania School of Medicine. Parents provided consent and children assent.

### Procedures

Families were recruited from general pediatric clinics as well as behavioral health clinics in the Philadelphia area. Phone screenings were conducted to verify initial inclusion criteria of age range between 6 and 18, presence of ADHD symptoms, Caucasian of European descent, availability and willingness to participate in a genetic study from both biological parents. Phone screenings also identified exclusionary criteria that included prematurity (< 36 weeks), mental retardation, major medical (excluding asthma) neurological disorders (e.g. seizures, fetal alcohol syndrome, plumbism) and neuropsychiatric disorders such as pervasive developmental disorder, psychoses, bipolar disorder, major depressive disorder with symptoms starting prior to ADHD or where ADHD symptoms occurred primarily during depressed episodes. All anxiety disorders were included. Children with documented IQ scores < 75 were excluded as were children with histories suggestive of mental retardation or inability to comprehend or complete the K-SADS.

Subjects who passed the phone screen proceeded with the K-SADS evaluation. Twelve subjects were excluded from the study after completing the diagnostic interview: Three subjects did not have impairing ADHD symptoms, 2 subjects met ADHD criteria primarily during a Major Depression and 2 subjects during a Generalized Anxiety Disorder, 1 subject with ADHD and Adjustment Disorder thought to significantly contribute to ADHD symptoms, 1 subject with ADHD and mild psychotic symptoms, 1 subject each with Cyclothymia, Bipolar Disorder and absence seizures. Siblings meeting inclusion and exclusion criteria were also invited to participate in the study, but their participation was not required.

### Measures

A child psychiatrist (JE) assessed diagnostic status by administering a K-SADS P-IVR interview to the parent(s) and child separately [9]. This semi-structured interview provides diagnoses occurring within the last twelve months of the present episode (PE) and for the last week (LW). It is keyed to the Research Diagnostic Criteria (RDC) [17] for those syndromes similar in youths and adults. All other diagnoses are DSM IIIR/IV [18] based and were made excluding the DSM diagnostic hierarchical requirements. The K-SADS-P IVR diagnostic domain is listed in Table 1. The primary diagnostician was trained in the K-SADS administration by a clinician (PJA) with extensive experience with this semi-structured interview. Intraclass correlations (ICC) between these raters for the diagnostic symptoms of the major disorders was assessed through videotape reviews. All ICC values were highly significant with the following ranges: affective disorders 0.74 – 0.85; anxiety disorder 0.75 – 0.92; ADHD: 0.82; ODD: 0.80.

**Table 1:** Diagnostic domain of K-SADS IVR

**Affective Disorders**	**Anxiety Disorders**	**Behavior Disorders**
Major Depressive	Avoidant Disorder(AD)	Oppositional Defiant Disorder(ODD)
Disorder(MDD)	Generalized Anxiety	Disruptive Behavior Disorder NOS
MDD, endogenous	Disorder(GAD)	
MDD, psychotic	Overanxious Disorder(OAD)	Attention Deficit-Hyperactivity
Irritable MDD	Post Traumatic Stress	Disorder(ADHD)
	Disorder(PTSD)	ADHD, without hyperactivity(ADD)
Minor Depression(MD)	Panic Disorder(PD)	ADHD, with hyperactivity(ADDH)
Irritable MD(IMD)	Separation Anxiety(SA)	ADHD, combined(ADDC)
Dysthymic Disorder(DD)	Simple Phobic Disorder(SPD)	ADHD, NOS(ADDN)
Irritable DD(IDD)	Social Phobia(SP)	
Depressive Disorder NOS	Obsessive Compulsive	Substance Abuse(SAB)
	Disorder(OCD)	Substance Dependence(SD)
Bipolar Depression, Mania	Anxiety Disorder NOS	
Hypomania		
Bipolar Depression		
Cyclothymia(CYCLO)		
Bipolar Depression NOS		

**Psychoses**	**Eating Disorders**	**Other Disorders**

Schizophrenia	Anorexia Nervosa	Schizotypal
Schizoaffective Disorder, depressed	Bulimic Disorder	Paranoid Disorder
	Eating Disorder NOS	Unspecified mental disorder
Schizoaffective Disorder, manic		
Unspecified functional psychosis		

**Adjustment Disorders**		

The K-SADS IVR records Irritability as a symptom independent of Depressed Mood. Irritability/Anger (subjective) was added as a third primary symptom of major depression to verify whether it was synonymous to depressed mood. This allows diagnosing an "Irritable Affective Disorder" which is a RDC or DSM defined depressive disorder where irritability replaces depressed mood or pervasive anhedonia as the cardinal symptom. For this study, Minor Depression (MD) and Dysthymic Disorder (DD) are combined into a variable labeled MDDD. Additional overall diagnostic variables were created for Any Anxiety Disorder (AnyAD) and for Any Depressive Disorder (AnyDep). The four main ADHD subtypes are labeled ADDI (Inattentive ADHD), ADDH (Hyperactive ADHD), ADDC (Combined ADHD), and ADDN (ADHD NOS).

Cognitive ability was quantified by reviewing reports of past IQ assessments. Children not previously tested were administered the Wechsler Abbreviated Scale of Intelligence (WASI). IQ scores are available for 241 subjects (70.5% of the sample). Socioeconomic status (SES) was calculated on 314 families (91.8%) by Hollingshead 4 factor scale [19]. Other pertinent data collected included proband prenatal and birth history, parental and teacher rated SWAN ADHD scales [20], and parental self report ADHD ratings. Bloods were also drawn from both parents and ADHD probands for genotyping.

### Data Analysis

Chi-square with continuity correction, Fisher exact probability, or binomial tests were used to analyze observed frequency or dichotomous data. Analysis of variance was used to test mean difference among continuous variables. All analyses were two tailed. Corrections for multiple comparisons were not made as the data analysis was exploratory in nature; significant p values were set equal to or less than 0.05. All analyses were done with SPSS 15.0.

## Results

The sample consists of 342 Caucasian patients all with a current diagnosis of ADHD. Within the sample there are 31 sibling pairs and 6 triple sibling groups comprising 62 and 18 patients respectively. This is approximately 24% of the sample. Twins or triplets were not included. Birth weight range was 5.5–12 lbs (mean 7.9 lbs; SD 1.1). IQ scores ranged from 77 to 147 (mean 110.2, SD 14.2; median 109.0). The mean SES raw score was 47.9 (SD 11.1) with a range from 18 to 66. The mean SES class was 2.0 (SD 0.9). None of these demographic parameters differed among the three main ADHD subtypes.

The subject's sex and age characteristics are shown in Table 2. Although there are significantly more boys (72%) than girls (28%) in each of the three main subtypes, their mean ages within each subtype are similar. The ADDI subjects were significantly older than those in the ADDH and ADDC groups regardless of sex (F = 8.9, df = 5, p < 0.00). The proportion of the three main ADHD subtypes was not evenly distributed. There was a greater percent of the Combined subtype (ADDC) (59.4%), than Inattentive subtype (ADDI) (31.0%) which also was more frequent than the Hyperactive subtype (ADDH) (9.1%). Two subjects (0.6%) were diagnosed with ADDN. More boys and fewer girls were in the ADDH group compared to the ADDI group (*x*^2 ^= 4.5, df = 2, p = 0.033). The sex ratios were not significantly different in other comparisons between the ADDC vs ADDH or the ADDC vs ADDI.

**Table 2:** Gender and age characteristics of ADHD sample

		**Male**	**Female**	**Total**	Sig
	n(%)	247(72.2)	95(27.8)	342	M > F, p=.000
**AGE:**					
**mean(sd)**		10.0(3.1)	10.6(3.3)	10.1(3.2)	Ns
**median**		9.3	9.9	9.4	
**range**		6.0–18.9	6.1–17.8	6.0–18.9	
**ADDC**	n(%)	149(73.4)	54(26.6)	203(59.4)	M > F, p = 0.000
**age(sd)**		9.4(2.7)	10.0(3.3)	9.6(2.9)	Ns
**ADDI**	n(%)	69(65.1)	37(34.9)	106(31.0)	M > F, p = 0.000
**age(sd)**		11.6(3.6)^1^	11.7(3.0)^1^	11.7(3.4)^1^	Ns
**ADDH**	n(%)	27(87.1)	4(12.9)	31(9.1)	M > F, p = 0.000
**age(sd)**		8.5(2.8)	8.2(1.5)	8.5(2.7)	Ns
**ADDN**	n(%)	2(100)		2(0.6)	
**age**		12.0		12.0	

ADDC: Combined type ADHD; ADDI: Inattentive ADHD; ADDH: Hyperactive ADHD;

ADDN: ADHD NOS; 1: sig. older than ADDC & ADDH (F = 21.7, df = 2, p < 0.00)

Tables 3, 4, 5 and 6 lists the concurrent co-morbid patterns within the sample. It only includes those diagnoses present. Diagnoses not present also reflect the clinical pattern of these subjects. In the Affective Disorders there were neither concurrent cases of endogenous, psychotic, irritable Major Depressive Disorder (MDD), nor any Depressive Disorder NOS. There were no cases of any Bipolar Disorder except Cyclothymia. There were no concurrent Eating Disorders or Anxiety Disorder NOS. All Behavior Disorders were present except Disruptive Behavior Disorder NOS. No Psychoses, Other Disorders, or Adjustment Disorders occurred concurrently with ADHD. Diagnostic patterns differed minimally between those present in the last year (PE) versus the last week (LW). Fifteen of 20 MDD subjects (8 recovered, 5 remitted to MDDD; 5 remained active; 1 had a recurrent MD, and 1 remitted to baseline DD) and 6 of 8 PTSD subjects were no longer fully symptomatic from the PE to the LW evaluation. As noted previously, actively depressed or manic patients were excluded at intake.

**Table 3:** Comorbid Anxiety Disorders in Affective Disorders^a^

**N(%)**	**MDD**	**MD**	**IMD**	**DD**	**IDD**	**MDDD**	**CYCLO**
**342**	**5(1.5)**	**31(9.1)**	**3(0.9)**	**36(10.5)**	**4(1.2)**	**74(21.6)**	**2(0.6)**
**AD 20(5.8)**	0	1(5.0)	1(5.0)	6(30.0)^1^	0	8(40.0)	0
**GAD 52(15.2)**	1(1.9)	8(15.4)	0	9(17.3)	2(3.8)	19(36.5)^1^	2(3.8)^1^
**PTSD 2(0.6)**	0	0	0	0	0	0	0
**PD 2(0.6)**	0	0	0	0	0	0	0
**SA 24(7.0)**	0	4(16.7)	0	2(8.3)	0	6(25.0)	0
**SPD 26(7.6)**	0	4(15.4)	0	4(15.4)	2(7.7)^1^	10(38.5)^1^	1(3.8)
**SP 9(2.6)**	0	0	0	2(22.2)	0	2(22.2)	0
**OCD 11(3.2)**	0	1(9.1)	0	1(9.1)	0	2(18.2)	0
**AnyAD 110(32.2)**	1(0.9)	14(12.7)	1(0.9)	16(14.5)	2(1.8)	33(30.0)^1^	2(1.8)

a. see Tb1 for diagnostic abbreviations; AnyAD: Any Anxiety Disorder;

1: significant co-occurrence, Fisher's Exact Test

**Table 4:** Comorbid Behavior Disorders in Affective Disorders^a^

**N(%)**	**MDD**	**MD**	**IMD**	**DD**	**IDD**	**MDDD**	**CYCLO**
**342**	**5(1.5)**	**31(9.1)**	**3(0.9)**	**36(10.5)**	**4(1.2)**	**74(21.6)**	**2(0.6)**
**ODD 139(40.6)**	4(2.9)	15(10.8)	3(2.2)	26(18.7)^1^	4(2.9)^1^	48(34.5)^1^	2(1.4)
**ADDI 106(31.0)**	1(0.9)	12(11.3)	0	10(9.4)	0	22(20.8)	1(0.9)
**ADDH 31(9.1)**	0	3(9.7)	0	3(9.7)	0	6(19.4)	0
**ADDC 203(59.4)**	4(2.0)	16(7.9)	3(1.5)	23(11.3)	4(2.0)	46(22.7)	1(0.5)
**ADDN 2(0.6)**	0	0	0	0	0	0	0
**CDSN 1(0.3)**	0	0	0	1(100)	0	1(100)	0
**SAB 6(1.8)**	0	1(16.7)	0	0	0	1(16.7)	0
**SD 3(0.9)**	0	0	0	1(33.3)	0	1(33.3)	0

a. see Tb1 for diagnostic abbreviations; MDDD: Minor Depression/Dysthymic Disorder;

1: significant co-occurrence, Fisher's Exact Test

**Table 5:** Comorbidity among Anxiety Disorders^a^

**N(%)**	**AD**	**GAD**	**PTSD**	**PD**	**SA**	**SPD**	**SP**	**OCD**
**342**	**20(5.8)**	**52(15.2)**	**2(0.6)**	**2(0.6)**	**24(7.0)**	**26(7.6)**	**9(2.6)**	**11(3.2)**
**AD 20(5.8)**	---	4(20.0)	0	0	0	1(5.0)	3(15.0)^1^	1(5.0)
**GAD 52(15.2)**	4(7.7)	---	0	1(1.9)	8(15.4)^1^	10(19.2)^1^	1(1.9)	2(3.8)
**PTSD 2(0.6)**	0	0	---	0	1(50.0)	0	0	0
**PD 2(0.6)**	0	1(50.0)	0	---	0	1(50.0)	0	0
**SA 24(7.0)**	0	8(33.3)^1^	1(4.2)	0	---	6(25.0)^1^	2(8.3)	0
**SPD 26(7.6)**	1(3.8)	10(38.5)^1^	0	1(3.8)	6(23.1)^1^	---	0	0
**SP 9(2.6)**	3(33.3)^1^	1(11.1)	0	0	2(22.2)	0	---	0
**OCD 11(3.2)**	1(9.1)	2(18.2)	0	0	0	0	0	---
**AnyAD 110(32.2)**	20(18.2)	52(47.3)	2(1.8)	2(1.8)	24(21.8)	26(23.6)	9(8.2)	11(10.0)

a. see Tb1 for diagnostic abbreviations; AnyAD: Any Anxiety Disorder;

1: significant co-occurrence, Fisher's Exact Test

**Table 6:** Comorbid Anxiety Disorders in Behavior Disorders^a^

**N(%)**	**ODD**	**ADDI**	**ADDH**	**ADDC**	**ADDN**	**CDSN**	**SAB**	**SD**
**342**	**139(40.6)**	**106(31.0)**	**31(9.1)**	**203(59.4)**	**2(0.6)**	**1(0.3)**	**6(1.8)**	**3(0.9)**
**AD 20(5.8)**	7(35.0)	9(45.0)	1(5.0)	10(50.0)	0	0	0	0
**GAD 52(15.2)**	24(46.2)	19(36.5)	7(13.5)	25(48.1)	1(1.9)	0	0	1(1.9)
**PTSD 2(0.6)**	2(100)	0	0	2(100)	0	0	1(50.0)^1^	1(50.0)^1^
**PD 2(0.6)**	1(50.0)	0	1(50.0)	1(50.0)	0	0	0	0
**SA 24(7.0)**	13(54.2)	5(20.8)	1(4.2)	18(75.0)	0	0	0	0
**SPD 26(7.6)**	16(61.5)^1^	4(15.4)	4(15.4)	17(65.4)	1(3.8)	0	0	0
**SP 9(2.6)**	3(33.3)	4(44.4)	0	5(55.6)	0	0	0	0
**OCD 11(3.2)**	2(18.2)	3(27.3)	1(9.1)	7(63.6)	0	0	0	0
**AnyAD 110(32.2)**	48(43.6)	36(32.7)	9(8.2)	63(57.3)	2(1.8)	0	1(0.9)	2(1.8)

a. see Tb1 for diagnostic abbreviations; AnyAD: Any Anxiety Disorder;

1: significant co-occurrence, Fisher's Exact Test

In the sample as a whole, the most prevalent co-occurring diagnoses were Oppositional Defiant Disorder (ODD:139, 40.6%), Minor Depression/Dysthmic Disorder (MDDD:74, 21.6%), and Generalized Anxiety Disorder (GAD:52, 15.2%). This pattern however varied when co-morbid patterns were considered in the three individual diagnostic groups (i.e., mood, anxiety, behavior). Among the MDDD group (n = 74), the most prevalent diagnoses were ODD (48, 64.9%), ADDC (46, 62.2%), and GAD (19, 25.7%). 32.2% of the total sample had at least one anxiety disorder. Among those with AnyAD (n = 110), the most common disorders were ADDC (63, 57.3%), GAD (52: 47.3%), and ODD (48: 43.6%). 30% (33) of the AnyAD group had MDDD. Those with ODD (n = 139) most commonly had ADDC (103: 74.1%), MDDD (48: 34.5%), and GAD (24: 17.4%). There were no significant sex differences in any of these diagnostic frequency patterns except that Separation Anxiety (SA) was more common in girls than boys (13.7% vs 4.5%, *x*^2 ^= 7.6, df = 1, p = .004).

Within the three main ADHD subtypes, the three most prevalent comorbid patterns also varied. In ADDI (n = 106), 20.8% had MDDD, 20.8% has ODD, and 18.6% had GAD. The most common diagnostic patterns in ADDH (n = 31) were ODD (41.9%), GAD (22.6%), and MDDD (19.4%). In ADDC (n = 203), the most prevalent disorders were ODD (50.7%), MDDD (22.7%), and GAD (12.3%). Within these three ADHD subtypes, MDDD and GAD were equally prevalent. However, ODD was significantly more prevalent in those with ADDC (50.7%) and ADDH (41.9%) compared to those with ADDI (20.8%). ODD was equally prevalent in those with ADDC and ADDH. Of all comorbid diagnoses, only ODD exhibited a different prevalence among the 3 ADHD subtypes.

The mean number of concurrent comorbid diagnoses in the total sample is 2.1, inclusive of ADHD. 36.3% (124) had only ADHD, 33.3% (114) had ADHD with one additional disorder, 18.7% (64) had two disorders, 8.2% (28) had three disorders, 2.9% (10) had four disorders, and two patients (0.6%) had five additional disorders besides ADHD. ODD occurred alone in 51.8% (59), GAD in 13.2% (15), and MDDD in 12.3% (14) of those with one additional diagnosis. 34.2% (39) only had AnyAD co-occurring with ADHD. In those with two or more additional diagnoses (n = 104), ODD occurred in 76.9% (80), MDDD in 57.7% (60), and GAD in 35.6%, (37). If Major Depressive Disorder (MDD) and Cyclothymia (CYCLO) are also included with the MDDD cohort, ODD and Any Affective Disorder co-occur in 54 cases which are 15.8% of the entire sample but represents 51.9% (54/104) of those with 3 or more diagnoses. All affective disorders (except Cyclothymia), GAD, SA and SPD were significantly more prevalent in those with 3 or more diagnoses compared to those with only 2 diagnoses. In the pure ADHD sample (124), the rate of ADDI was 35.5%(44), ADDH 9.7%(12), and ADDC 54.8%(68). This proportional pattern of ADHD subtypes was similar to that of the remaining group with 2 or more diagnoses.

Subjects with Panic Disorder (PD) were the youngest (mean age 8.0) but there were only 2 cases. Subjects with SA and ADDH were the next youngest diagnostic groups (mean age 8.5). The oldest diagnostic group was the Substance Abuse Disorder/Substance Dependence (SAB/SD) patients (mean age 16.5). Patients with SA or ADDH were significantly younger than those patients without these diagnoses. Those with SP, ADDI, ADDC, and SAB/SD were significantly older than those without these diagnoses. There were no significant age differences between the boys and girls in any diagnostic group except for the boys with SP being older than the girls with SP (12.6 vs 10.2). However, there were only two girls with SP. The ADDH group were significantly younger than either the ADDC or ADDI subjects (F = 21.7, df = 2, p = 0.00).

Given the high prevalence of ODD, the sample was dichotomized into those with or without ODD and comorbid characteristics reanalyzed. Those subjects with ODD were significantly more likely to have MDDD, SPD, ADDC and significantly less likely to have ADDI. Within the Affective Disorder group, the significant correlation with ODD was predominantly driven by DD, IDD, and IMD. 64.9% (48/74) of MDDD subjects had ODD versus 34.0% of those without MDDD (x^2 ^= 21.7, df = 1, p = 0.00).

Because the symptom of Irritability has taken center stage in the comorbid diagnostic pattern of ADHD and Bipolar Disorder in preadolescent children, its comorbid characteristics were analyzed in this sample. 66 subjects (81.8% prepubertal; 18.2% adolescent) either had subjective (from the Depressive K-SADS module) or overt (from the Mania module) Irritability. This represents 19.3% of the entire sample of ADHD subjects. Only 7 subjects had concurrent subjective and overt Irritability. Irritability was significantly associated with all of the Affective Disorder subtypes. In the Anxiety Disorder group, it was significantly associated with Avoidant Disorder; and, in the Behavior Disorder group it was significantly associated with ODD and ADDC. Within the Irritable group, 66.7% (44/66) have MDDD, 71.2% (47/66) have ADDC, and 80.3% (53/66) have ODD. This specific comorbid triad (i.e., MDDD, ODD, ADDC) represents 10.8% (37/342) of the entire sample. 31 of these 37 cases (83.8%) are those with symptomatic Irritability. These 31 cases represent almost half of those with Irritability (31/66, 47.0%).

## Discussion

Although the comorbid frequencies in this report do not reflect a naturalistic sample, these diagnostic patterns are quite similar to prior clinical studies reflecting diagnoses endorsed by both child and parent informants. This data set however includes the broadest age range and the most extensive description of concurrent comorbidity of any study which is based on summary data from both the parent and child regardless of the subject's age.

Over 1/3^rd ^of cases had pure ADHD. These findings are similar to those reported in the MTA study, based on parental DISC reports, in a comparable clinical cohort which reported a 31.8%, 6 month prevalence rate of pure ADHD, although from a younger age (mean age 8.5 years; range 7.0 to 9.9) and mixed racial sample of only ADDC subjects [10]. The proportion of pure ADHD cases within the ADDC sample from this study was 33.5% (68/203). Our data does diverge from a report in a general Swedish school age population that suggested that pure ADHD was rare [21]. This stands out as increased rates of comorbidity are expected in a clinic sample such as this. However, the Swedish group had a small sample of ADHD subjects, including children with various developmental disabilities who were excluded from this study.

ADDH represents less than 10% of the total sample of ADHD subjects. This rate is lower than reported epidemiological frequencies yet ADDH was still the least prevalent of the three subtypes in such samples [22,23]. In a clinical outpatient sample assessing lifetime comorbidity from Biederman's group [24], the ADHD subtype frequencies were almost identical to those reported in this study (ADDC: 61% vs 59%; ADDI: 30% vs 31%; ADDC: 9% vs 9%, respectively). The age pattern was also similar as the ADDI were the oldest, the ADDH the youngest while the ADDC were intermediate in age. The male/female ratio was approximately 3 to 1 in each study for the total sample, ADDI and ADDC.

ADDH and ADDC are dominated by ODD with a 49.6% (116/234) combined comorbidity rate while ADDI has a more uniform comorbid pattern among affective, anxious, and behavior syndromes. ODD pervasiveness in ADDH/ADDC is further seen as it is the overwhelming comorbidity occurring in nearly 70% (51/74) of those with only one additional diagnosis. Affective and Anxious disorders only co-occur as a second diagnosis in approximately 6.8% and 23.0% respectively of the combined ADDH/ADDC subtypes with one additional diagnosis. These co-occurrence patterns between ADHD and affective, anxious and behavior disorders partially validate Angold's analysis [1] which noted a more highly significant odds ratio pairing between ADHD and CD/ODD than ADHD and Depressive or ADHD and Anxiety disorders. His analysis however did not differentiate among the ADHD subtypes and analyzed epidemiological samples. The pairing of affective, anxious and behavior disorders significantly differed between the ADD and ADDH/ADDC in this clinical sample since AnyAD (*x*^2 ^= 9.6, df = 1, p = 0.002,) and AnyDep (*x*^2 ^= 4.9, df = 1, p = 0.028) were likely paired with ADD while ODD (*x*^2 ^= 22.1, df = 1, p = 0.00) was more likely paired with ADDH/ADDC.

The high co-occurrence of an affective disorder with ADHD is reported in numerous settings [25,26] and in a 5 year prospective outcome study of ADHD girls [7], yet most of these studies refer to life time or prospective co-occurrence. The high prevalence of concurrent MDDD in this cohort is significant particularly since actively depressed cases were excluded from this study as was low birth weight which in itself could account for higher rates of depression in girls [27]. It suggests that Minor Depression and Dysthmia may be a more significant morbid factor in the disability of ADHD. The higher prevalence of affective disturbances in females was not noted here but this pattern emerges by the early teen years which is older than the mean age of this sample [28].

The finding that Irritability as a symptom was highly associated with MDDD and ODD is not surprising since it is part of the diagnostic domain of Affective Disorders and ODD [29]. Angold et al have shown the significant co-occurrence of these diagnoses with ADHD [1]. However what seems unique here is the greater likelihood of it occurring in the preadolescent child linked with a diagnostic triad of MDDD, ODD, and ADDC. This finding compliments the previously reported link of Irritability with ODD, MDDD and ADHD in a group of depressed and anxious adolescents [30]. Vance et al. [31] have also noted that ADDC and DD significantly predicted ODD symptomatology in a sample of preadolescent ADDC children chosen to exclude comorbid MDD.

In the present sample of 342 subjects, Irritability occurred in 20% (54/272) of the preadolescents and in 17% (12/70) of the adolescent subjects. However within the Irritable subjects themselves, 81.8% (54/66) were preadolescents. It may be that it is this concurrent comorbid triad with irritability is being over diagnosed as the prepubertal Bipolar child. This data set's exclusion of Bipolarity only adds more weight to this hypothesis. Whether this subset of ADHD patients represents an enriched future Bipolar core can only be evaluated through follow-up studies. Nonetheless, there are current reports identifying ODD and ADHD are more prevalent in youths with Irritable Bipolar NOS [32].

### Limitations

This clinical sample does not represent the general population of clinically referred ADHD youths since the study required the participation of both biological parents and it was limited to Caucasians. Its comorbid patterns could diverge from ADHD youths growing up in less-intact families as well as ethnically mixed ADHD samples. However, this approach was necessitated by the primary genetic aims of this study. The young mean age also implies that many subjects have not passed through syndrome specific age of onset time frames. This was most notable in the same sex prevalence of MDDD. The sample also excluded obvious active cases of both Major Depressive and Bipolar disorders so it can not further elucidate characteristics of the Bipolar/ADHD debate.

The majority of the K-SADS IVR interviews were completed by one rater which could introduce an information collection bias to this data set. Also, the K-SADS does not diagnoses learning disorders which can be co-morbid in 20–25% of ADHD subjects [33].

### Clinical Implications

Almost 2/3^rd ^of ADHD children have at least one additional impairing diagnosis. It is all too easy to overlook this pattern and focus treatment solely on ADHD. As noted in the MTA study, specific treatment combinations may be more efficacious in certain comorbid patterns [10]. It also is clear that ADDC is the most prevalent ADHD subtype in clinical cohorts while ADDI is the most prevalent in epidemiological samples [22,23].

The comorbidities described here reflect that occurring within a selected ADHD sample chosen for a genetic analysis. It would be inappropriate therefore to interpret these rates suggesting they are "true" comorbid patterns in other disorders since the rate of ADHD is 100% within every diagnosis recorded in this data set.

The clinician should be aware that Minor Depression/Dysthymia is common in ADHD and not be quick to dismiss dysphoric states as adverse events of stimulant treatment or the demoralization of having a significantly debilitating illness.

The concurrent comorbid pattern displayed by this ADHD cohort generally corresponds with previously reported ADHD clinical samples. This suggests this data set is sufficiently representative of the ADHD clinical population to serve as a valid sample for genetic analysis. Concurrent comorbid analysis of the ADHD population may allow for a more comprehensive understanding of the vicissitude of this syndrome and assist in identifying potential endophenotypes. Phenotyping data from this study has been submitted to the NIMH repository. Genotyping data will also be submitted upon completion of study.

## Competing interests

The authors declare that they have no competing interests.

## Authors' contributions

JE participated in study design, subject recruitment, phenotype ascertainment, data analyses and interpretation. PJA participated in study design, data analyses and interpretation. WB participated in study design, data analyses and interpretation. All authors read and approved the final manuscript.
